# Social networks, social support and psychiatric symptoms: social determinants and associations within a multicultural community population

**DOI:** 10.1007/s00127-014-0943-8

**Published:** 2014-08-09

**Authors:** Natasha Smyth, Chesmal Siriwardhana, Matthew Hotopf, Stephani L. Hatch

**Affiliations:** 1 Department of Psychological Medicine, Institute of Psychiatry, King’s College London, De Cresigny Park, Denmark Hill, London, SE5 8AF UK; 2 Health Services and Population Research Department, Institute of Psychiatry, King’s College London, De Cresigny Park, Denmark Hill, London, SE5 8AF UK; 3 Department of Psychological Medicine, Weston Education Centre, Institute of Psychiatry, King’s College London, Cutcombe Rd, London, SE5 8RJ UK; 5 Present Address: Institute of Neurology, UCL, 12 Queen Square, London, WC1N 3BG UK

**Keywords:** Social support, Social networks, Mental health, London, UK

## Abstract

**Purpose:**

Little is known about how social networks and social support are distributed within diverse communities and how different types of each are associated with a range of psychiatric symptoms. This study aims to address such shortcomings by: (1) describing the demographic and socioeconomic characteristics of social networks and social support in a multicultural population and (2) examining how each is associated with multiple mental health outcomes.

**Methods:**

Data is drawn from the South East London Community Health Study; a cross-sectional study of 1,698 adults conducted between 2008 and 2010.

**Results:**

The findings demonstrate variation in social networks and social support by socio-demographic factors. Ethnic minority groups reported larger family networks but less perceived instrumental support. Older individuals and migrant groups reported lower levels of particular network and support types. Individuals from lower socioeconomic groups tended to report less social networks and support across the indicators measured. Perceived emotional and instrumental support, family and friend network size emerged as protective factors for common mental disorder, personality dysfunction and psychotic experiences. In contrast, both social networks and social support appear less relevant for hazardous alcohol use.

**Conclusions:**

The findings both confirm established knowledge that social networks and social support exert differential effects on mental health and furthermore suggest that the particular type of social support may be important. In contrast, different types of social network appear to impact upon poor mental health in a more uniform way. Future psychosocial strategies promoting mental health should consider which social groups are vulnerable to reduced social networks and poor social support and which diagnostic groups may benefit most.

## Introduction

Apparent in the literature is a reasonable consensus that research into the social relationships and affiliation of an individual involves understanding the extent of their involvement and attachment to others through public and private interactions [1]. Conceptually, the social networks of an individual are considered in relation to their ability to access various types of social support in their everyday lives [2]. More specifically, social networks encompass the structure of social ties, which vary in source and frequency. In comparison, social support refers to the social resources that individuals perceive as available or have received, which functions to serve a variety of needs, whether emotional or instrumental in nature [3]. Whilst social networks and social support are therefore theoretically distinct, it is likely that social support is contingent upon the presence of social networks and that the two components constitute a multi-level continuum. According to Lin et al. [2], individuals are embedded within a multi-layered support system, with their structural social network environment serving to enhance various functional aspects of social support. Although it cannot be assumed that every social network contact will automatically lead to social support, the two components are inevitably linked. Since Durkheim’s seminal work linking suicide with social integration and cohesion [4], a large body of theoretical and empirical work has sought to demonstrate and explain the broadly protective effects of social networks and social support on mental health [5–8]. However, much related research has been hampered by a number of methodological shortcomings.

The conceptualisation and operationalisation of social networks and social support have varied across studies, limiting the comparability of results generated. Social networks and social support are often used interchangeably as concepts despite theoretical recognition that they should be treated distinctively, albeit with some inevitable overlap [3]. Some recent studies show a failure to distinguish between social networks and social support within analysis, despite the potential to do so with the measurement tools used [9, 10]. Whilst other studies only investigate specific types of social networks and thus only offer a narrow insight into the structural aspects of social relationships, rather than the function they serve for the individual [11]. This is significant because social networks and social support are not only known to exert differential effects on mental health outcomes [2, 12] but are also likely to have impact via different pathways [1]. It has been suggested that whilst not mutually exclusive, social support may be more important for individuals under stress, operating via the ‘stress-buffering model’, but social networks may have a beneficial effect in all circumstances, operating via ‘the main effects model’ [1].

Despite early articulation of the need to also consider social network and social support measures as dependent variables [6, 13], focus on how these resources vary across different population subgroups, according to source and type, has been limited [14]. However, understanding the conditions which may constrain or enable social networks and social support is necessary for the development of effective interventions proposing either or both as mental health-promoting resource [1].

Another issue is the limited effort towards a more comprehensive inclusion of contextualising variables. While most research accounts for age and gender as socio-demographic characteristics [2, 15], there has been a notable absence of other potentially important contextualising variables, such as migrant status and ethnicity. Given the disruption that migrants often experience to their social networks [16], and recent findings suggesting variation in specific mental health outcomes by ethnicity [17, 18], these socio-demographic factors should be considered. Indeed where ethnicity and migration status have been explored regarding perceived support from family and friends, differences have been identified. For example, a neighbourhood study in Chicago found that compared to non-Latino whites, Latino participants reported higher family support, and all ethnic minority groups reported lower perceived friend support [14]. Another study that examined amalgamated measures of social network and social support found that the adverse effect of low support was the strongest amongst more recent migrants [10]. Where used, community samples often lack ethnic diversity [2] and/or are restricted to particular age, gender or occupation cohorts [14, 19, 20].

Finally, research investigating how social networks and social support may be associated with mental health has often relied on crude indicators (e.g. receipt of a mental illness diagnosis in the past year [10] or self-rated mental health as excellent, very good, good, fair or poor [9]). Studies commonly include measures of ‘general psychiatric distress’ [21, 22] or focus on a single psychiatric disorder, usually depression [2, 23, 24]. In response, Chou et al. [11] used data from a national US survey to examine social isolation (i.e. the absence of frequently contacted close friends and religious group affiliates) in relation to multiple mood, anxiety and substance use disorders, and demonstrated that social isolation exerted a differential impact by type of disorder. Infrequent religious contacts were positively associated with substance use disorders, whilst infrequent close friend contact was positively associated with major depressive disorder, social phobia, dysthymic disorder and generalised anxiety disorder. Little research has been carried out to explicitly test the association between social networks or social support and mental health outcomes, such as personality dysfunction and psychotic symptoms. The former is recognised to be associated with impaired social functioning, with previous research demonstrating an increased likelihood of reporting single relationship status [25, 26] and smaller social networks in comparison to those suffering from other psychiatric disorders [27]. In relation to the latter, previous research has found an association between social isolation and suffering from first-episode psychosis [28].

This study aims to address several shortcomings in this research area by utilising measures of both social networks and social support and examining their association with mental health in an ethnically and economically diverse inner city community sample. The specific aims are to: (1) examine the distribution of social network and social support indicators by socio-demographic and socioeconomic factors and (2) investigate the relationship between social networks, social support and multiple mental health outcomes. Given the variation found in previous research [11, 12], we hypothesise that the pattern of association between these measures and mental health outcomes will vary. More specifically, we expect that disorders characterised by internalising symptoms will show a different relationship to social networks and social support components than those characterised by externalising symptoms.

## Method

### Sample

Data for this study were collected between 2008 and 2010 as part of the South East London Community Health Study (SELCoH) [17, 29]; a cross-sectional psychiatric and physical morbidity survey of 1,698 adults, aged 16 or over, residing in the South London boroughs of Lambeth and Southwark. These boroughs represent areas of high deprivation compared to the national average, but also encompass areas of relative wealth [30, 31].

Participants were recruited from 1,075 randomly selected households using the Small User Postcode Address File (PAF) which ensures near complete coverage of private UK households. Comparisons with the 2011 UK Census data for the study catchment area indicated that the sample was largely representative regarding key demographic and socioeconomic indicators. A computer-assisted interview schedule was used by trained interviewers to carry out face-to-face interviews. This study was approved by the King’s College London research ethics committee. See [17] for a detailed description of the study methodology.

## Measurement

### Social network indicators

Social networks refer to the type and size of participants’ social networks [2]. Type and size of network indicators were generated from reports of contact with people (face to face or by phone) in a typical week. Social networks were characterised as the cumulative number of contacts by the following types: (1) family social network and (2) friend social network (including close friends, neighbours, other acquaintances, member of same group or club).

### Social support indicators

To assess social support, participants were asked whether they hypothetically have someone to: (1) lend them money to pay bills or help them get along; (2) help with an emergency (minor or health emergency); (3) talk to when something was bothering them or when they felt lonely and wanted company and (4) make them feel good, loved or cared for. The first two items were combined to capture sources of ‘perceived instrumental support’, whilst the latter two were combined as sources of ‘perceived emotional support.’ Scores ranged between 0 and 2 for each indicator.

### Mental health outcomes

A range of internalising and externalising symptoms are included as mental health outcomes. Common mental disorder (CMD) was assessed using the Revised Clinical Interview Schedule (CIS-R) [32]. The CIS-R is an interviewer administered structured set of questions asking about 14 symptom domains including depression and anxiety. A total CIS-R score of 12 or more is conventionally used to indicate the overall presence of CMD. Through a standard algorithm, the CIS-R also provides ICD-10 diagnoses for other mental disorders, including generalised anxiety disorder and depressive episodes.

Hazardous alcohol use was measured using the World Health Organization Alcohol Use Disorders Identification Test (AUDIT) [33]. The AUDIT includes 10 questions exploring patterns of consumption, symptoms of alcohol dependence and problems associated with alcohol misuse in the past year. The participant receives an overall score ranging between 0 and 40 with a cut-point of 8 or more indicating caseness.

Personality dysfunction was assessed using the Standardised Assessment of Personality—Abbreviated Scale [34]. This involves eight dichotomously rated descriptive statements about the person, with final scores ranging between 0 and 8. In clinical populations, a score of 3 or more is used to indicate the presence of personality dysfunction, however, here a cut-point of 4 was used. This gives a slightly improved positive predictive value, as the prevalence in a community population is assumed to be lower [35].

Psychotic symptoms were assessed using the Psychosis Screening Questionnaire (PSQ) [36]. This assesses five symptom domains: hypomania, thought insertion, paranoia, strange experiences and hallucinations. This outcome represents endorsement of at least one symptom, with the exception of hypomania which was omitted due to a particularly high positive response rate.

### Socio-demographic and socioeconomic indicators

Socio-demographic indicators included gender, ethnicity, age, migration status and household composition. Participants reported their ethnicity according to the following groups: White, Black Caribbean, Black African, Black Other, Indian, Pakistani, Bangladeshi, Chinese and Other. The categories were collapsed into White, Black Caribbean, Black African and Other to improve distribution across groups. Age (in years) was categorised into the following groups: 16–30, 31–45, 46–60, 60 or above. Migrant status indicated whether or not the participant was born in the UK and length of time in the UK in the following categories: UK born; 0–4 years; 5–10 years; 11 years or more. For household composition, a categorical indicator of ‘living with others’ and ‘living with others/single parent’ was created.

Socioeconomic status included educational attainment, employment status and household income. Educational attainment was condensed into two categories: up to GCSE level (no qualifications and qualifications up to GCSE or Ordinary level, i.e. high school equivalent) and A Level or above (up to Advanced level and university degree level qualification or above). The first group encompasses compulsory education, whilst the second group encompasses non-compulsory education and represents the necessary route to higher education. Employment status was categorised as follows: (1) employed (full time, part time or casual work); (2) unemployed; (3) full time or working student and (4) other (temporary and permanent sick/disabled, retired or looking after the home with children). It should be noted that the majority of students in the sample was working.

### Statistical analysis

All analysis was conducted using STATA 11 (StataCorp, 2008). Survey commands (svy) were used for the estimates of prevalence and associations to generate robust standard errors. All analyses accounted for clustering by household inherent in the study design and weighted for within household non-response, comparing all eligible household members (i.e. 16 years or older) by gender and age (see further details in [17]). Unweighted frequencies and weighted percentages are presented for all study variables. Following this, unadjusted associations between socio-demographic and socioeconomic factors and social network and social support indicators were assessed using ordered logistic regression, with the exception of household network for which multinomial logistic regression was used. Logistic regression was used to explore associations between the social network and social support indicators and all mental health outcomes, for which social network and social support variables were dichotomised. Social network indicators were recategorised to indicate either none or some weekly contact. Social support variables were recategorised to indicate either low support (combining the reporting of neither or only one of the items), or high support. This decision was made in recognition that the absence of either of the associated items represents a fairly fundamental deficit in perceived support. For this analysis the following models were estimated: model 1 is unadjusted; model 2 adjusts for potential socio-demographic confounders; model 3 further adjusts for all potential socioeconomic confounders and model 4 further adjusts for all social network and social support indicators. Given the potential overlap across the mental health outcomes, we have corrected for multiple comparisons with the Bonferroni method and adopted a more conservative significance level of 0.0125 for all mental health models. All available cases were used within the analysis [17, 29]. Consistent with previous studies [9, 37], the social network and social support variables were tested for collinearity and found to be moderately correlated. Thus, an alternative strategy was investigated whereby these variables were removed from models following full attenuation of associations to avoid one variable masking the effect of another. However, this approach was not required given the minimal change to the strength of associations present.

## Results

### Sample description

Table 1 presents the characteristics of the study participants. The sample is diverse in terms of representation of ethnic minority groups (36.5 %) and migration status (40.3 % born outside of the UK). CMD was the most prevalent outcome (24.2 %), while psychotic-like symptoms (9.7 %) were less common. The vast majority of participants had some weekly contact with friends (95.2 %) and family (91.6 %). The majority reported having both types of instrumental (82.4 %) and emotional support (89.7 %).

**Table 1:** Sample characteristics of the South East London Community Health Study (SELCoH)

	Total sample *n* (%)		Total sample *n* (%)
Total sample	1, 698	Common mental disorder	
Gender		No	1,296 (75.8)
Female	959 (66.7)	Yes	396 (24.2)
Male	739 (33.3)	Hazardous alcohol use	
Age (years)		No	1,344 (82.5)
16–30	622 (30.7)	Yes	343 (17.5)
31–45	504 (26.8)	Personality dysfunction	
46–59	339 (21.9)	No	1,421 (84.7)
60+	233 (20.6)	Yes	241 (15.3)
Ethnic group		Psychotic symptoms	
White	1,051 (63.5)	No	1,402 (83.9)
Black Caribbean	143 (8.7)	Yes	285 (16.1)
Black African	234 (13.2)	Family social network	
Other	268 (14.7)	No contact	149 (8.4)
Migration status		1 contact	518 (31.9)
UK born	1,005 (59.7)	2 contacts	785 (45.7)
0–4 years	139 (6.9)	3 contacts	232 (14.0)
5–10 years	184 (10.0)	Friend social network	
≥11 years	361 (23.4)	No contact	78 (4.8)
Household composition		1 contact	344 (20.3)
Living with others	1,327 (74.1)	2 contacts	516 (30.3)
Living alone/single parent	371 (25.9)	3 contacts	515 (30.1)
Educational attainment		4 contacts	231 (14.5)
Up to GCSE level	560 (37.1)	Perceived instrumental support	
A level or above	1,119 (63.0)	No support	64 (4.3)
Employment status		1 support	217 (13.3)
Employed	921 (51.2)	2 supports	1,396 (82.4)
Unemployed	170 (9.3)	Perceived emotional support	
Student	247 (12.5)	No support	48 (2.9)
Other	351 (27.1)	1 support	116 (7.4)
		2 supports	1,512 (89.7)

Percentages are weighted to account for survey design; frequencies are unweighted and may not add up due to missing values

### Characteristics of social networks and social support indicators

Table 2 identifies groups at risk of reduced social networks and poor social support by documenting unadjusted associations for each indicator by socio-demographic and socioeconomic factors. Family network size did not differ greatly by these covariates, with the exception of Black African and other ethnic groups reporting larger family networks compared to the White group and those living with others reporting larger family networks than those in the living alone/single parent group. In comparison, friend network size was associated with all covariates aside from gender and household composition. Notably, migrants residing in the UK for 10 years or less reported smaller friend networks than the reference group. Lower educational attainment groups reported smaller friend networks than those with more education. Similarly, smaller friend networks were reported by those in the unemployed group compared to those employed.

**Table 2:** Unadjusted associations between social network indicators, social support indicators and individual covariates

	Family network size	Friend network size	Instrumental support	Emotional support
	Regression coefficient (95 % CI) *p*	Regression coefficient (95 % CI) *p*	Regression coefficient (95 % CI) *p*	Regression coefficient (95 % CI) *p*
Gender				
Male	Ref	Ref	Ref	Ref
Female	0.21 (0.03, 0.40) *p* = 0.025	−0.15 (−0.32, 0.02) *p* = 0.090	0.06 (−0.20, 0.33) *p* = 0.635	0.37 (0.03, 0.70) *p* = 0.032
Age (years)				
16–30	Ref	Ref	Ref	Ref
31–45	0.02 (−0.21, 0.25) *p* = 0.858	−0.08 (−0.13, 0.30) *p* = 0.449	−0.45 (−0.79, −0.12) *p* = 0.008	−0.11 (−0.60, 0.38) *p* = 0.663
46–59	−0.11 (−0.37, 0.16) *p* = 0.425	−0.40 (0.15, 0.65) *p* = 0.002	−0.70 (−1.07, −0.33) *p* ≤ 0.001	−0.85 (−1.31, −0.38) *p* ≤ 0.001
60+	−0.30 (−0.61, 0.01) *p* = 0.055	−0.26 (−0.03, 0.55) *p* = 0.079	−0.92 (−1.33, −0.51) *p* ≤ 0.001	−0.88 (−1.39, −0.38) *p* = 0.001
Ethnic group				
White	Ref	Ref	Ref	Ref
Black Caribbean	0.22 (−0.09, 0.54) *p* = 0.167	−0.02 (−0.34, 0.30) *p* = 0.902	−0.66 (−1.14, −0.17) *p* = 0.008	−0.20 (−0.81, 0.41) *p* = 0.526
Black African	0.59 (0.31, 0.87) *p* ≤ 0.001	−0.01 (−0.31, 0.28) *p* = 0.933	−1.17 (−1.52, −0.81) *p* ≤ 0.001	−0.11 (−0.58, 0.35) *p* = 0.637
Other^a^	0.49 (0.20, 0.78) *p* = 0.001	−0.41 (−0.66, −0.16) *p* = 0.001	−0.55 (−0.93, −0.17) *p* = 0.004	−0.08 (−0.54, 0.39) *p* = 0.748
Migration status				
UK born	Ref	Ref	Ref	Ref
0–4 years	−0.07 (−0.45, 0.31) *p* = 0.710	−0.63 (−0.94, −0.33) *p* ≤ 0.001	−0.36 (−0.85, 0.12) *p* = 0.140	−0.12 (−0.64, 0.89) *p* = 0.751
5–10 years	0.14 (−0.17, 0.46) *p* = 0.374	−0.52 (−0.80, −0.25) *p* ≤ 0.001	−1.07 (−1.47, −0.67) *p* ≤ 0.001	0.36 (−0.89, 0.17) *p* = 0.184
≥11 years	0.32 (0.05, 0.59) *p* = 0.020	−0.09 (−0.33, 0.15) *p* = 0.472	−0.84 (−1.18, −0.51) *p* ≤ 0.001	−0.37 (−0.77, 0.02) *p* = 0.065
Household composition				
Living with others	Ref	Ref	Ref	Ref
Living alone/single parent	−0.55 (−0.89, −0.31) *p* ≤ 0.001	−0.24 (−0.46, −0.24) *p* = 0.030	−0.30 (−0.61, 0.07) *p* = 0.056	−0.73 (−1.09, −0.36) *p* ≤ 0.001
Educational attainment				
Up to GCSE level	−0.02 (−0.22, 0.18) *p* = 0.848	−0.45 (−1.65, −0.25) *p* ≤ 0.001	−0.87 (−1.15, −0.58) *p* ≤ 0.001	−0.90 (−1.26, −0.54) *p* ≤ 0.001
A level or above	Ref	Ref	Ref	Ref
Employment status				
Employed	Ref	Ref	Ref	Ref
Unemployed	−0.16 (−0.49, 0.18) *p* = 0.363	−0.53 (−0.84, −0.23) *p* = 0.001	−0.90 (−1.32, −0.49) *p* ≤ 0.001	−1.13 (−1.63, −0.64) *p* ≤ 0.001
Student	−0.07 (−0.31, 0.16) *p* = 0.548	0.02 (−0.20, 0.24) *p* = 0.843	0.59 (0.05, 1.12) *p* = 0.032	0.40 (−0.25, 1.05) *p* = 0.230
Other	−0.19 (−0.44, 0.07) *p* = 0.151	−0.26 (−0.51, −0.02) *p* = 0.032	−0.87 (−1.19, −0.54) *p* ≤ 0.001	−0.77 (−1.18, −0.36) *p* ≤ 0.001
Family network size	–	–	0.29 (0.12, 0,46) *p* = 0.001	0.48 (0.27, 0.70) *p* ≤ 0.001
Friend network size	–	–	0.39 (0.25, 0.52) *p* ≤ 0.001	0.63 (0.47, 0.80) *p* ≤ 0.001

*Ref* reference group

^a^Other includes all Asian or mixed groups

As with social network contact, no difference was found in social support level by gender. Across most age groups, being older was associated with less instrumental and emotional support. Ethnic minority groups reported less instrumental support than the white ethnic group, as did migrants who had resided in the UK for 5 years or more compared to those UK born. In comparison, no difference was found for emotional support by ethnicity or migrant status. Those living alone or as single parents reported less emotional support compared to those living with others, but no difference was found in instrumental support. Lower educational attainment was associated with less instrumental and emotional support. Similarly, less instrumental and emotional support was reported by those in the unemployed and ‘other’ employment status groups. Both family and friend social networks were positively associated with both types of social support.

### Social networks, social support and mental health

Table 3 describes associations between social network and social support indicators and mental health outcomes. Having a high level of emotional support was associated with decreased odds of CMD after all adjustments. Additional adjustments for all social network and social support indicators attenuated associations between weekly family and friend contact, instrumental support and CMD. Additional analysis of the two most prevalent primary diagnoses generated by the CIS-R, generalised anxiety disorder and depressive episodes, indicated no association between any of the social network or support indicators and generalised anxiety disorder. In contrast, having weekly family and friend contact and a high level of emotional support were associated with decreased odds of depressive episodes after all adjustments. An initial association found between instrumental support and decreased odds of depressive episodes was attenuated by the inclusion of socioeconomic indicators.

**Table 3:** Mental health outcomes by social network and social support indicators

	Model 1	Model 2	Model 3	Model 4
	Unadjusted odds ratio (95 % CI) *p*	Adjusted odds ratio (95 % CI) *p*	Adjusted odds ratio (95 % CI) *p*	Adjusted odds ratio (95 % CI) *p*
Common mental disorder—CIS-R (12+)				
Family network size	0.59 (0.41, 0.86) *p* = 0.005	0.55 (0.38, 0.81) *p* = 0.003	0.52 (0.36, 0.77) *p* = 0.001	0.60 (0.40, 0.90) *p* = 0.013
Friend network size	0.48 (0.29, 0.79) *p* = 0.004	0.42 (0.25, 0.69) *p* = 0.001	0.50 (0.29, 0.86) *p* = 0.012	0.64 (0.36, 1.14) *p* = 0.130
Instrumental support	0.58 (0.43, 0.78) *p* ≤ 0.001	0.49 (0.36, 0.67) *p* ≤ 0.001	0.54 (0.39, 0.75) *p* ≤ 0.001	0.74 (0.52, 1.07) *p* = 0.106
Emotional support	0.37 (0.27, 0.53) *p* ≤ 0.001	0.33 (0.23, 0.48) *p* ≤ 0.001	0.33 (0.23, 0.48) *p* ≤ 0.001	0.42 (0.28, 0.62) *p* ≤ 0.001
Hazardous alcohol use				
Family network size	0.64 (0.43, 0.96) *p* = 0.032	0.70 (0.45, 1.09) *p* = 0.112	0.73 (0.46, 1.15) *p* = 0.169	0.70 (0.44, 1.12) *p* = 0.139
Friend network size	2.10 (1.02, 4.34) *p* = 0.045	1.82 (0.83, 4.01) *p* = 0.133	1.65 (0.75, 3.63) *p* = 0.217	1.54 (0.69, 3.44) *p* = 0.288
Instrumental support	2.28 (1.50, 3.46) *p* ≤ 0.001	1.38 (0.88, 2.15) *p* = 0.162	1.28 (0.81, 2.01) *p* = 0.292	1.24 (0.78, 1.96) *p* = 0.356
Emotional support	1.44 (0.88, 2.36) *p* = 0.146	1.11 (0.63, 1.94) *p* = 0.723	1.10 (0.61, 1.97) *p* = 0.755	0.99 (0.53, 1.85) *p* = 0.983
Personality dysfunction				
Family network size	0.43 (0.28, 0.65) *p* ≤ 0.001	0.41 (0.27, 0.64) *p* ≤ 0.001	0.36 (0.23, 0.57) *p* ≤ 0.001	0.38 (0.24, 0.61) *p* ≤ 0.001
Friend network size	0.32 (0.19, 0.54) *p* ≤ 0.001	0.28 (0.16, 0.47) *p* ≤ 0.001	0.31 (0.17, 0.56) *p* ≤ 0.001	0.41 (0.22, 0.76) *p* = 0.005
Instrumental support	0.43 (0.31, 0.60) *p* ≤ 0.001	0.39 (0.27, 0.57) *p* ≤ 0.001	0.47 (0.32, 0.69) *p* ≤ 0.001	0.71 (0.46, 1.09) *p* = 0.119
Emotional support	0.31 (0.21, 0.46) *p* ≤ 0.001	0.29 (0.19, 0.43) *p* ≤ 0.001	0.31 (0.20, 0.47) *p* ≤ 0.001	0.41 (0.25, 0.66) *p* ≤ 0.001
Psychotic symptoms				
Family network size	0.71 (0.46, 1.10) *p* = 0.127	0.68 (0.43, 1.08) *p* = 0.101	0.98 (0.50, 1.91) *p* = 0.954	1.14 (0.56, 2.32) *p* = 0.714
Friend network size	0.96 (0.52, 1.78) *p* = 0.903	0.83 (0.44, 1.56) *p* = 0.567	0.98 (0.43, 2.19) *p* = 0.952	1.42 (0.62, 3.25) *p* = 0.407
Instrumental support	0.58 (0.42, 0.81) *p* = 0.001	0.55 (0.38, 0.78) *p* = 0.001	0.44 (0.28, 0.68) *p* ≤ 0.001	0.53 (0.33, 0.83) *p* = 0.006
Emotional support	0.37 (0.26, 0.54) *p* ≤ 0.001	0.32 (0.22, 0.48) *p* ≤ 0.001	0.35 (0.21, 0.59) *p* ≤ 0.001	0.43 (0.25, 0.74) *p* = 0.002

Model 1 unadjusted, model 2 adjusted for gender, age, ethnicity, migration status, household composition, model 3 adjusted for gender, age, ethnicity, migration status, household composition, education, employment, model 4 adjusted for gender, age, ethnicity, migration status, household composition, education, employment and all social network and social support variables

Fully adjusted model sample sizes are as follows: Common Mental Disorder (*n* = 1,625) hazardous alcohol use (*n* = 1,623); personality dysfunction (*n* = 1,611); psychotic symptoms (*n* = 1,627)

In contrast to the CMD findings, no association was found between any of the social network or social support variables and hazardous alcohol use in the final models. Socio-demographic characteristics attenuated an initial association with instrumental support, whilst no association was found with family or friend contact and emotional support even within the unadjusted model. Having weekly contact with family and friends as well as a high level of emotional support was associated with decreased odds of personality dysfunction in the fully adjusted models. In contrast, associations with low instrumental support were attenuated in the final model. Finally, having increased emotional and instrumental support was associated with decreased odds of psychotic-like symptoms in the fully adjusted model. In contrast, neither of the social network indicators were associated with this outcome.

## Discussion

Drawing from a diverse inner city community sample, considerable variation was found in social networks and social support by demographic characteristics. In comparison, a pattern emerged demonstrating reduced social networks and social support among socioeconomically disadvantaged individuals. Regarding mental health outcomes, emotional social support emerged as a particularly important for mental health, in contrast instrumental support appeared less important as a protective factor across a range of mental health outcomes. Neither social networks nor social support appears to be related to hazardous alcohol use. These results not only confirm established knowledge that social networks and social support exert differential impact on poor mental health but go further to highlight that the particular type of social support is of importance. In contrast, different types of social network appear to share a more similar pattern of association with poor mental health.

The results demonstrate that the distribution of social network and social support by socio-demographic factors can be better understood by including commonly omitted factors, such as ethnicity and migrant status. Consistent with the limited evidence available [9, 14], differences in family and friend networks were found between some ethnic minority groups compared to the white majority group. In addition, all ethnic minority groups reported less instrumental support than the white majority group. Migrant status also appeared important; compared to those UK born, being a migrant was associated with decreased friend networks and less perceived instrumental support. Whilst a largely consistent pattern was found linking socioeconomically deprived individuals with decreased levels of social networks and social support, the size of this effect varied depending on the SES measure and type of network or support indicator. Our findings suggest that those reporting less education, for example, may be particularly vulnerable to a decreased levels of emotional support. However, it is likely that there are specific profiles combining demographic and SES indicators, such as recent migrant status and being out of employment that could usefully inform psychosocial related interventions. By understanding the distribution of social networks and social support at the community level, higher risk groups can be identified.

Consistent with other studies exploring social network and social support in relation to multiple mental health outcomes [11, 12], our findings suggest that there is a need to consider differential effects of distinct social support components on specific mental health symptoms. It was found here that perceived emotional support was more important than instrumental support for mental health. This distinction may be all the more important if it is still the case that perceived support constitutes the most frequently assessed support-related construct [38], and widespread belief persists that overall it is significantly more related to emotional well-being than other types of support or network components [21]. It may be that some mental disorders (e.g. hazardous alcohol misuse) have little or no relationship with social networks or social support. However, any relationship is likely to be complex; it may be that whilst alcohol misuse tends to occur in a social space, for some it may lead to social isolation. Equally, difficulty in making and retaining friendships is a recognised symptom of personality dysfunction; thus, the strong association found with weekly contact with friends may be harder to disentangle. It should be noted that each of the mental health outcomes tested here have clinically different aetiology and profile and that it is beyond the scope of this study to draw deeper conclusions about the exact nature of these relationships. Furthermore, what has been measured here are levels of psychiatric symptoms within a community sample rather than clinical diagnosis of psychiatric disorder. It is possible that individuals within the sample may have scored above the threshold for more than one mental disorder, however this has been corrected for through the adoption of a more conservative significance level. These findings have important implications for the future design of related psychosocial interventions. As previously stated, the baseline characteristics, such as interpersonal skills, of individuals most likely to benefit from such interventions need to be better elucidated [7]. The findings here suggest that it may also be important to further understand which diagnostic groups are more likely to benefit.

In acknowledging the study limitations, the cross-sectional design of the study prevents the ability to determine the direction of effect or make casual inferences about the associations found. Smaller social networks or a lower level of perceived support may increase the risk of poor mental health but equally, the presence of mental health symptoms may disrupt or limit an individual’s ability to sustain network ties or perceive available support. The non-response rates at the individual level in the national study and at the household level in the SELCoH sample may have resulted in participation bias so the prevalence estimates should be considered with caution. However, the SELCoH sample was shown to be representative on most demographic and socioeconomic characteristics of the population in the study catchment area according to the UK Census [17]. Despite these limitations this study provides rich, descriptive community data; such samples are not typical of the support-related literature which more typically includes community samples lacking in social and cultural diversity [2, 14, 19, 20], or clinical populations [39, 40] with limited generalisability for general community populations. Further, this study explores a wider range of social network and support indicators and mental health outcomes than many previous studies.

Overall, these findings highlight the important relationship that exists between particular types of social network and social support indicators and specific mental health disorders. The results reinforce the need for research to continue to recognise the distinction between structural network and functional support. By exploring a range of mental health outcomes, we see that rather than impacting in a uniform way, the pattern of association with social networks and social support components varies. There is a clear implication for the effective delivery of support-related interventions; consideration should be given to the manner in which social relationships are being enhanced and the diagnostic group of recipients. Furthermore the findings demonstrate the varied social distribution of these resources by a range of characteristics, including ethnicity and migrant status which are not commonly accounted for. Future research should aim to include such contextualising variables and interventions should consider targeting social groups likely to be at a higher risk of reduced networks and poor support. For example, given the broadly consistent pattern found linking socioeconomically deprived individuals with decreased levels of both, service providers working with individuals engaging in job seeking or those not currently in training or education should also focus resources on finding ways to reduce social isolation.
